# Telehealth as a Resource for Continuity of Care in the Face of Disaster

**DOI:** 10.1093/geroni/igab046.1796

**Published:** 2021-12-17

**Authors:** Walter Boot, Judith Robertson Phillips

## Abstract

This symposium co-sponsored by the Disasters and Older Adults and the Technology and Aging special interest groups of GSA aims to highlight the promise of, and barriers to, the use of telehealth to support continuity of care in the face of disasters and crises, such as the ongoing COVID-19 pandemic. M. Mattos will showcase a home-based medical care (HBPC) program to address chronically ill and homebound persons living with dementia and caregivers’ needs during the pandemic. T. Wyte-Lake will present the results of a national survey on how changes were made to the Department of Veterans Affairs (VA) HBPC programs in response to the pandemic. G. Demiris describes a large caregiver study in which problem solving therapy and positive appraisal theory interventions designed specifically to support family caregivers of hospice patients during the COVID-19 pandemic were implemented via telehealth. D. Lindeman will specifically discuss challenges and implementation strategies for telehealth solutions applied to low-income older adults living in affordable housing communities. Finally, H. Xu will present the results of an analysis examining the effectiveness of telehealth in reducing readmissions among heart failure patients during the COVID-19 pandemic. While the COVID-19 pandemic has especially impacted older adults and those who care for them, these talks highlight the potential of telehealth services and interventions to provide support and facilitate the continuity of care during times of crisis.

